# Sustainable Materials with Improved Biodegradability and Toughness from Blends of Poly(Lactic Acid), Pineapple Stem Starch and Modified Natural Rubber

**DOI:** 10.3390/polym16020232

**Published:** 2024-01-14

**Authors:** Wasan Tessanan, Pranee Phinyocheep, Taweechai Amornsakchai

**Affiliations:** 1 Department of Chemistry, Faculty of Science, Mahidol University, Rama VI Road, Payathai, Bangkok 10400, Thailand; t.wasan18@gmail.com (W.T.); pranee.phi@mahidol.ac.th (P.P.); 2 Center of Sustainable Energy and Green Materials, Faculty of Science, Mahidol University, Phuttamonthon 4 Road, Salaya, Nakhon Pathom 73170, Thailand; 3 TEAnity Team Co., Ltd., 40/494 Soi Navamintra 111, Khet Bueng Kum, Bangkok 10230, Thailand

**Keywords:** poly(lactic acid), pineapple stem starch, agricultural waste, modified natural rubber, sustainable resource, toughening agent

## Abstract

Poly(lactic acid) (PLA), derived from renewable resources, plays a significant role in the global biodegradable plastic market. However, its widespread adoption faces challenges, including high brittleness, hydrophobicity, limited biodegradability, and higher costs compared to traditional petroleum-based plastics. This study addresses these challenges by incorporating thermoplastic pineapple stem starch (TPSS) and modified natural rubber (MNR) into PLA blends. TPSS, derived from pineapple stem waste, is employed to enhance hydrophilicity, biodegradability, and reduce costs. While the addition of TPSS (10 to 40 wt.%) marginally lowered mechanical properties due to poor interfacial interaction with PLA, the inclusion of MNR (1 to 10 wt.%) in the PLA/20TPSS blend significantly improved stretchability and impact strength, resulting in suitable modulus (1.3 to 1.7 GPa) and mechanical strength (32 to 52 MPa) for diverse applications. The presence of 7 wt.% MNR increased impact strength by 90% compared to neat PLA. The ternary blend exhibited a heterogeneous morphology with enhanced interfacial adhesion, confirmed by microfibrils and a rough texture on the fracture surface. Additionally, a downward shift in PLA’s glass transition temperature (T_g_) by 5–6 °C indicated improved compatibility between components. Remarkably, the PLA ternary blends demonstrated superior water resistance and proper biodegradability compared to binary blends. These findings highlight the potential of bio-based plastics, such as PLA blends with TPSS and MNR, to contribute to sustainable economic models and reduce environmental impact for using in plastic packaging applications.

## 1. Introduction

In recent decades, the accumulation of vast amounts of plastic waste has emerged as a pressing environmental crisis and one of the most severe threats humanity faces. Strict environmental regulations on the use of traditional non-degradable plastics have been implemented globally, aligning with the UN’s goals and encompassing the three pillars of sustainable economics: the Bio-Circular-Green (BCG) economy [1]. Among the various alternative approaches, recycling and the development of biodegradable or compostable plastics have garnered significant attention as essential strategies for addressing these environmental crises. Utilizing biodegradable plastics presents promising avenues to optimize and conclude the life cycle of conventional non-degradable plastic products [2,3]. Numerous types of biodegradable or compostable polymers have been developed and are commercially available, including fully biobased poly(lactic acid) (PLA), partially biobased poly(butylene succinate) (PBS), and fully synthetic biodegradable polymers such as poly(ε-caprolactone) (PCL) and poly(butylene adipate-co-terephthalate) (PBAT). Additionally, other biopolymers, such as starch-based plastics and polyhydroxyalkanoates (PHAs), have found applications in various industries [4,5]. PLA, a linear aliphatic polyester derived from renewable resources like corn, wheat, sugarcane, and rice, stands out as a leading product segment in the global biodegradable plastic market. Its ease of processability using conventional equipment, biocompatibility, high mechanical strength, and environmental friendliness have made it a preferred choice in packaging, agriculture, textile, and biomedical devices [6]. However, the extensive use of PLA is hindered by major drawbacks, including high brittleness, hydrophobicity, low degradation rate, and relatively high cost compared to traditional petroleum-based plastics, thereby limiting its market potential. Consequently, numerous researchers have made substantial efforts to address these disadvantages [7,8,9].

A polymer blend and composite system represent a straightforward and efficient approach to achieving desired material properties. To enhance the ductility, reduce costs, and accelerate the degradation rate of PLA, various low-cost additives have been proposed, including calcium carbonate [10], biochar [11], wood powder [12], natural fiber [13,14], nanoparticles [15,16], phenolic compound [16], vegetable oils [17], and other biopolymers like natural rubber [18,19] and starch-based plastic [20,21]. Thermoplastic starch, owing to its low cost, renewability, biodegradability, ease of handling, and availability, has emerged as a promising candidate to complement PLA’s limitations [22]. However, the mechanical properties of PLA/thermoplastic starch blends, especially mechanical strength, stretchability and impact strength, often suffer due to insufficient interfacial interaction and phase adhesion arising from differences in polarity between PLA and thermoplastic starch, leading to the incompatibility of PLA and thermoplastic starch as the dispersed phase, resulting in a phase separation of the PLA/thermoplastic starch blend system [20,21,23,24]. Addressing this issue, additives such as acrylic acid [25] and methylene diphenyl diisocyanate [26], have been employed to enhance blend miscibility. Moreover, the introduction of rubbery polymers as a third component, such as poly(ethylene-co-octene)-graft-(glycidyl methacrylate) [27] and ethylene-co-vinyl acetate copolymer [24], into the relatively brittle PLA/thermoplastic starch blend has shown promise in improving toughness and other properties. Despite these advancements, most substances used to enhance PLA/thermoplastic starch blend properties are synthetic materials. Consequently, the development of PLA/thermoplastic starch blends toughened with renewable materials, aiming to attain the desired properties, remains a scarce area in scientific research and continues to pose a significant emerging challenge. Natural rubber (NR) and its derivatives have been explored for this purpose [7,28]. NR, commonly known as a green elastomeric material primarily composed of cis-1,4-polyisoprene derived from the Hevea brasiliensis tree, has found widespread use in various applications due to its abundant availability, cost-effectiveness, renewability, high elasticity, and ease of chemical modification and biodegradability. Diverse forms of modified NR, including hydrogenated NR, epoxidized NR, maleated NR, and acrylated NR, have been developed for a range of applications [29]. Using natural rubber in polymer blends and composites was reported with the proper physical and mechanical properties, as well as biodegradability [30,31]. Nevertheless, there has been limited scientific research on the utilization of NR-based materials for toughening the PLA/thermoplastic starch blend system, highlighting a significant gap in the existing body of knowledge.

Therefore, our current research aims to develop a PLA/thermoplastic pineapple stem starch (TPSS) blend system and enhance the blends using modified natural rubber (MNR) through a melt blending procedure employing an internal mixer. Pineapple stem starch (PSS), a substantial agricultural waste derived from pineapple stems after bromelain extraction, with approximately 34% amylose content [32], was processed into thermoplastic pineapple stem starch using an internal mixer. In a previous study, Shi et al. [27] prepared thermoplastic starch from corn starch (17–25% amylose content) and blended with the PLA using the melt-blending process. The result found that the PLA/thermoplastic starch (80/20) binary blend has mechanical strength and impact strength of approximately 37 MPa and 5 kJ/m^2^. Nazrin et al. [20] extracted the sugar palm starch (~38% amylose content) from sugar palm trees and prepared thermoplastic starch reinforced with sugar palm nanocellulose fiber using solution casting technique before melt-blending with PLA in an internal mixer. As a result, the PLA/thermoplastic starch (80/20) reinforced with 0.5% sugar palm nanocellulose fiber had a modulus of 1.2 GPa and mechanical strength of 20 MPa. Fonseca-García et al. [21] utilized achira starch (~29% amylose content) to prepare thermoplastic starch and blended it with PLA using the solution casting method. The findings reveal that the PLA/thermoplastic starch (75/25) blend film is highly brittle, without further investigation of the mechanical properties. Due to the substantial amylose content in PSS, we hypothesize that TPSS, with its inherent hydrophilicity and biodegradability, could offer superior mechanical properties in the PLA/TPSS blend compared to other starch types. The natural hydrophilic and biodegradable characteristics of TPSS may significantly complement the properties of the PLA/TPSS blend. Modified natural-rubber-containing hydroxyl functional groups serve as the toughening agent in the PLA/TPSS blend system. The vital rationale for using MNR in this research is an eco-friendly and facile synthesis with one-step preparation and cost-effectiveness of reagent compared to other modification pathways. The hydroxyl group of this typical modified rubber is expected to form the physical and chemical interactions with the blended system. Therefore, we aim to study the impact of TPSS contents (10, 20, 30, and 40 wt.%) and MNR contents (1, 3, 5, 7, and 10 wt.%) on mechanical, thermal, morphological, dynamic mechanical properties, water resistance, and degradation behaviors, with the aim of advancing our understanding and optimizing the performance of this innovative blend.

## 2. Materials and Methods

### 2.1. Materials

Poly(lactic acid) or PLA (Ingeo™ Biopolymer 2003D, NatureWorks LLC, Minnetonka, MN, USA) with a density of 1.24 g/cm^3^ and melt flow index (210 °C, 2.16 kg) of 6 g/10 min was procured through local distributor. Pineapple stem starch (PSS) was prepared from pineapple stem waste, an agricultural biomass residue of the bromelain extraction, and was supplied by Hong Mao Biochemical Co., Ltd. (Rayong, Thailand). High-ammonia NR latex (60% dried rubber content) was supplied by Thai Rubber Latex Co., Ltd. (Samut Prakan, Thailand). Hydrogen peroxide (30%) was purchased from Carlo Erba Reagent (Milan, Italy). Tergitol 15-S-15 was procured from Dow Inc. (Midland, MI, USA). Additionally, commercial-grade glycerol was acquired from a local supplier.

### 2.2. Preparation of Modified Natural Rubber (MNR)

**Figure 1:**
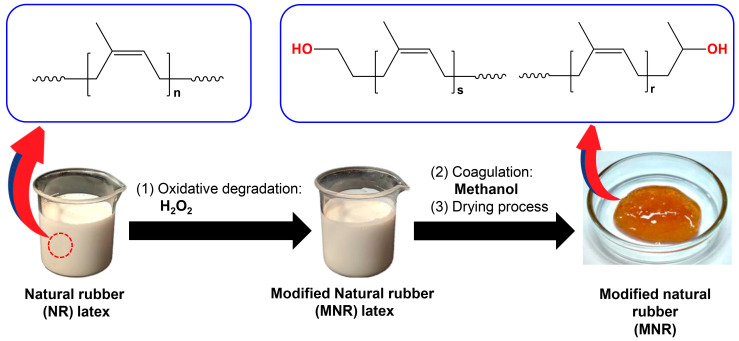
Schematic preparation of modified natural rubber (MNR).

MNR was prepared through a facile and eco-friendly process using natural rubber latex as a starting material. The schematic modification of NR to prepare MNR in the latex stage is demonstrated in Figure 1. Firstly, 100 mL (60 g of solid rubber) of 60% dried rubber content of high-ammonia NR latex was weighed and diluted to a 20% dried rubber content of NR latex by adding distilled water (200 mL). Afterwards, 3 phr of Tergitol 15-s-15 as a non-ionic surfactant was added into the rubber latex and stirred for 24 h to stabilize the rubber–colloidal system. Subsequently, 100 mL of hydrogen peroxide (1 mol) was slowly added to the diluted NR latex through a separating funnel at 60 °C and stirred for 12 h. At the end of the reaction, the MNR latex was coagulated with methanol and washed with water several times to remove the residue reagents. The coagulated rubber was then dried in a hot oven at 60 °C for seven days to receive the yellowish-orange dried rubber before characterization.

### 2.3. Preparation of Thermoplastic Pineapple Stem Starch (TPSS)

**Figure 2:**
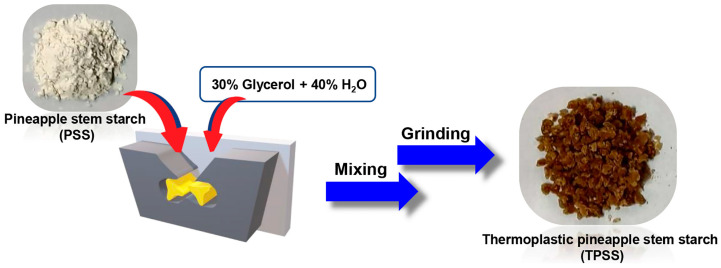
Schematic preparation of thermoplastic pineapple stem starch (TPSS).

Firstly, 50 g of pineapple stem starch (PSS) powder was blended with 30% glycerol and 40% water based on the weight of the starch using a high-speed mixer (28,000 rpm) for 2 min. Subsequently, the starch mixture was stored for 24 h before gelatinization in the internal mixer equipment with a rotor speed of 100 rpm for 3 min at 170 °C. The obtained thermoplastic pineapple stem starch (TPSS) was then ground using a grinder machine to receive the TPSS granules. Figure 2 demonstrates the schematic preparation of TPSS.

### 2.4. Preparation of Thermoplastic Pineapple Stem Starch (TPSS)

The PLA, TPSS, and MNR were dried in a vacuum oven at 80 °C for 24 h before the blending process to remove the humidity. Subsequently, the PLA pellets were melt-blended with TPSS and MNR in an internal mixer (Haake^TM^ Rheomix 90, Thermo Fisher Scientific, Waltham, MA, USA) at 170 °C for 15 min with a rotor speed of 50 rpm according to previous work [33]. The formulation of PLA/TPSS blends and PLA/TPSS blends toughened by MNR is shown in Table 1. Afterwards, the PLA blends were pulverized by a grinder machine to acquire the PLA blend granules. Eventually, the PLA blend granules in each formulation were dried in a vacuum oven at 40 °C for 48 h before the compression process at 170 °C for 2 min to prepare the specimen for further testing. 

### 2.5. Material Characterizations

#### 2.5.1. Determination of Hydroxyl Value of MNR

The hydroxyl value of MNR was determined with alkalinity correction using a titration approach according to ASTM D4274 [34]. Approximately 1 g of MNR was added to a 250 mL Erlenmeyer flask and dissolved in 25 mL of phthalic anhydride solution using pyridine as a solvent. Subsequently, the rubber solution was heated at 155 °C for 1 h and then reduced in temperature to room temperature (25 ± 2 °C). Then, 50 mL of pyridine was added to the rubber solution before adding 0.5 mL of phenolphthalein (1% *w/v* in pyridine) as an indicator. The mixture solution was titrated using 0.5 N sodium hydroxide solution and the end point of the titration process was evaluated when the color of the mixture solution changed from colorless to pink without the color change within 30 s. The blank solution was also carried out without the addition of rubber. The hydroxyl value of MNR was calculated using Equation (1).

Hydroxyl number (mg KOH/g) = ((B − A) × N/*w*) × 56.1 (1)

where A and B are the volume of sodium hydroxide used for titration of MNR solution and blank solution (mL). N is the normality of sodium hydroxide solution. *w* is the weight of MNR (g). The calculation of the hydroxyl value of MNR was performed in triplicate and the mean and standard deviation values were demonstrated.

#### 2.5.2. Fourier Transform Infrared (FTIR) Spectroscopy

The attenuated total reflection accessory equipped with Fourier transform infrared (ATR-FTIR) spectra of MNR and PLA blends were acquired from an infrared spectrophotometer (Paragon 1000, PerkinElmer, Waltham, MA, USA). The spectra were assessed in the range of 4000 cm^−1^ to 400 cm^−1^ with 64 scans and a resolution of 4 cm^−1^.

#### 2.5.3. Proton Nuclear Magnetic Resonance (^1^H-NMR) Spectroscopy

The chemical structure of MNR was confirmed using a Ultrashield N.M.R. spectrometer with a 500 MHz operating frequency (Bruker Corporation, Karlsruhe, Germany). Approximately 10 mg of MNR was completely dissolved at room temperature in deuterated chloroform (CDCl_3_) as a solvent and tetramethylsilane (TMS) as an internal standard. The chemical shifts are expressed in part per million (ppm). The CDCl_3_ solvent peak is 7.26 ppm, and the TMS peak is 0.0 ppm.

#### 2.5.4. Gel Permeation Chromatography (GPC)

The molecular weight of the MNR was determined via gel permeation chromatography (GPC) (150-C ALC/GPC, Waters, Milford, MA, USA). Approximately 10 mg of rubber samples were dissolved in tetrahydrofuran (THF) as a solvent and filtered through a 13 µm nylon syringe filter with a 0.45 µm pore size before analysis. THF as an eluent was manipulated with a constant flow rate of 1 mL/min at 40 °C. The number average molecular weight (M_n_), the weight average molecular weight (M_w_), and the polydispersity index (PDI) of the rubber samples were obtained in this measurement.

#### 2.5.5. Mechanical Properties

The tensile behavior of the PLA blends was investigated according to ASTM D882 [35] using a universal tensile testing machine (Instron 5566, Instron, High Wycombe, UK). Ten rectangular thin sheets of each sample were measured with a 50 mm/min crosshead speed and a 1 kN static load cell capacity at room temperature (25 ± 2 °C). The mean and standard deviation of tensile properties, including Young’s modulus, tensile strength, and strain at break, were reported.

The impact resistance of the PLA materials was investigated by conforming to ASTM D256 [36] using a pendulum impact tester machine (Zwick/Roell HIT5.5P, Ulm, Germany). Six rectangular specimens were notched with a Zwick/Roell manual notch cutting machine and tests were carried out with an Izod configuration at room temperature (25 ± 2 °C). The mean and standard deviation values are expressed in the unit of J/m.

The hardness of the PLA blends was assessed following ASTM D2240 [37] using a hardness Shore D tester (GT-GS-MB model, Hardness Tester Gotech Testing Machines, Taichung City, Taiwan). The mean and standard deviation values of each sample were derived from six-specimen testing.

#### 2.5.6. Dynamic Mechanical Analysis

The viscoelastic properties of the neat PLA and PLA blends were considered using a dynamic mechanical analyzer (Q800, TA Instrument, New Castle, DE, USA). Investigation of a rectangular specimen of each sample with a dimension of 7 mm × 30 mm × 2 mm (width × length × thickness) was carried out under tension mode using a constant frequency of 10 Hz and a heating rate of 2 °C/min. The scanning temperature proceeded ranging between −80 °C and 100 °C. The storage modulus (G′) and tan δ plotted against temperature were acquired.

#### 2.5.7. Morphological Properties

The impact-fractured and the cryo-fractured surfaces of the sample were observed using a scanning electron microscope (SEM) (JSM-IT500, JEOL, Tokyo, Japan). Before the SEM analysis, the samples were uniformly coated with platinum as an ultra-thin film of electrically conducting metal.

A three-dimensional (3D) topographic color map of the impact-fractured specimen was visualized using an optical 3D surface profiler (TopMap Pro. Surf+, Polytec, Waldbronn, Germany). The impact-fractured specimen was placed on the stage of equipment before observation. The measurement was performed using TMS software (v4.2) for operating the surface scanning parameters with different reflections and contrast in each sample.

#### 2.5.8. Thermal Properties

The thermal transition behavior of the samples was assessed using a differential scanning calorimeter (DSC) (Q200-RCS90, TA Instrument, New Castle, DE, USA). The blends were performed from 30 °C to 200 °C with a heating rate of 10 °C/min. The glass transition temperature (T_g_), cold crystallization temperature (T_cc_), and melting temperature (T_m_) were reported, and the degree of crystallinity (*X*_c_) was determined using Equation (2).

Degree of crystallinity (*X*_c_) = ((ΔH_m_ − ΔH_cc_)/(*w*_PLA_ × ΔH°_m_)) × 100 (2)

where ΔH_cc_ and ΔH_m_ are the enthalpy of cold crystallization and melting of the PLA blends, respectively. ΔH°_m_ is the enthalpy of PLA at 100% crystallinity (93.7 J/g) [38]. *w*_PLA_ is the weight fraction of PLA in the blend system.

#### 2.5.9. Water Absorption

The water-resistant ability of the PLA blend samples was assessed in terms of water absorption, following the modified approach reported previously by Namphonsane et al. [39]. The sample sheets with a dimension of 20 mm × 20 mm (width × length) were dried in a hot-air oven at 80 °C for 24 h to remove the humidity in the samples and the dried weight was recorded, which was defined as *w*_i_. Subsequently, the sample sheet was soaked and gently stirred in distilled water (pH = 7). The absorption ability was measured at different time intervals (1, 2, 3, 4, 5, 6, 7, 15, and 30 days) of immersion time at room temperature (25 ± 2 °C). Excess water on the sample surface was removed using blotting paper, and its weight was recorded and is represented as *w*_f_. Water absorption was determined using Equation (3). The calculation of water absorption was carried out in triplicate, and the mean and standard deviation values were reported.

Water absorption (%) = ((*w*_f_ − *w*_i_)/*w*_i_) × 100 (3)

#### 2.5.10. Soil Burial Degradation Test

The biodegradability of samples was evaluated using a soil burial test, following a modified procedure previously ascribed by Seligra et al. [40]. Sample sheets with dimensions of 20 mm × 20 mm (length × width) were shaped and placed within high-density polyethylene envelopes. These envelopes were buried around 10 cm below the soil surface at the garden edge adjacent to the department building, with a soil pH of 7.5. The test area was shaded by trees and received regular watering. The degradation rate of the sample was visually investigated, and data were collected at specified time intervals throughout three months. The samples in each time interval were wiped with deionized water and dried in a hot air oven at 60 °C for 24 h before the weight of the plastic sheets in each sample after the soil burial test was assessed. The biodegradable level was determined using Equation (4) [23]. Each sample’s mean and standard deviation values at different time intervals were reported.

Biodegradable rate (%) = ((*w*_0_ − *w*_t_)/*w*_0_) × 100 (4)

where *w*_0_ is the original weight of the sample sheet before the soil burial test and *w*_t_ is the weight of the sample after the soil burial test at different time intervals.

## 3. Results and Discussion

### 3.1. Chemical Structure, Hydroxyl Content, and Molecular Weight of Modified Natural Rubber

**Figure 3:**
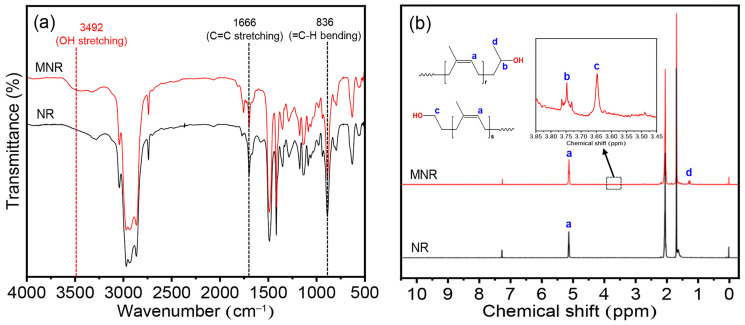
Chemical structure: (**a**) ATR-FTIR and (**b**) ^1^H-NMR spectra of NR and MNR.

The intricate chemical structure of the modified NR (MNR) was assessed using an attenuated total reflection-Fourier transform infrared (ATR-FTIR) and proton nuclear magnetic resonance (^1^H-NMR) analysis, as shown in Figure 3. The absorption spectrum of NR as a starting material (Figure 3a) demonstrates prominent vibrational peaks at 1666 cm^–1^ and 836 cm^–1^, associated with the double bond of the isoprene unit of NR. Other vibrational peaks at around 2800–3000 cm^–1^ and at about 1445 and 1375 cm^–1^ correspond to C-H stretching and C-H bending vibrations, respectively [41]. The modification of NR using hydrogen peroxide as an oxidizing agent in the latex stage displays a new absorption peak at 3492 cm^–1^, assigned to the O-H stretching vibration, involving the formation of hydroxyl groups along the NR backbone after the oxidative cleavage degradation reaction by hydrogen peroxide [42]. Subsequently, the chemical structure of MNR was also asserted using the ^1^H-NMR technique, as seen in Figure 3b. The unique signal of NR appeared at 5.14 ppm (-CH_3_C=C**H**-, a), corresponding to the methine proton adjacent to the isoprene unit of NR. After the oxidative cleavage reaction, the obtained MNR showed similar signals with the starting NR material and three new signals with small intensity were observed at 1.28 ppm (-C**H_3_**CH-OH, d), 3.65 ppm (-C**H_2_**-OH, c), and 3.75 ppm (-CH_3_C**H**-OH, b), associated with the proton adjacent to the hydroxyl group [43].

**Figure 4:**
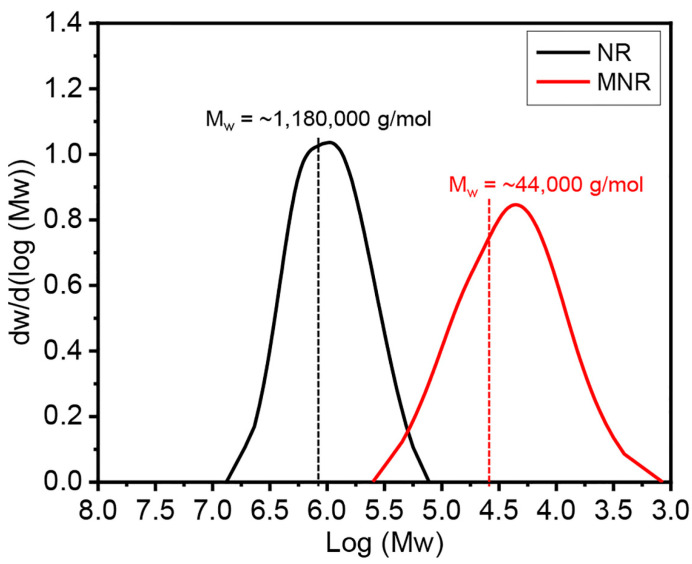
Molecular weight distribution curves and the weight average molecular weights of NR and MNR.

The hydroxyl value of MNR was determined using a titration method according to ASTM D4274 [34]. The calculated result of the hydroxyl value of the MNR was 30.57 ± 2.03 mg KOH/g. Subsequently, the molecular weight of the synthesized MNR was measured using the gel permeation chromatography (GPC) technique, and the distribution curves of the molecular weight of rubber are demonstrated in Figure 4. The finding results reveal that NR has number average molecular weight (M_n_) and the weight average molecular weight (M_w_) values of approximately 950,000 g/mol and 1,180,000 g/mol, respectively, with a polydispersity index (PDI) of 1.24. The obtained MNR had a molecular weight of approximately 44,000 g/mol (M_n_) and 15,000 g/mol (M_w_) with a PDI of 2.93. As a result, it can be seen that the modified rubber obtained in this work consisted of the hydroxyl functional group with low molecular weight, resulting in the formation of a light amber sticky rubber-like material, as seen in Figure 1, similar to the previous report by Ibrahim et al. [42].

### 3.2. Properties of the Blends

#### 3.2.1. Mechanical Properties

**Figure 5:**
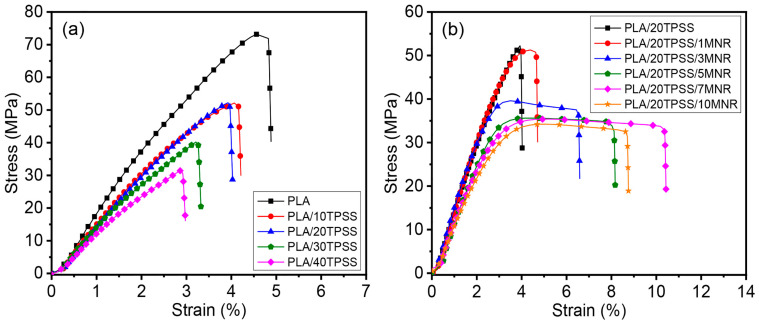
Stress–strain curves of (**a**) PLA/TPSS binary blends with various TPSS contents and (**b**) PLA/20TPSS/MNR ternary blends with various MNR contents.

Figure 5 exhibits the stress–strain curves of the PLA blends with various thermoplastic pineapple stem starch (TPSS) and modified NR (MNR) contents. The relevant values are summarized in Table 2. The neat PLA shows the typical rigid and brittle aspect with an abrupt breakage without yield point involving high tensile modulus (2.14 GPa) and high tensile strength (73.18 MPa) with low stretchability (4.84%). Nevertheless, adding TPSS displays a drastic decline in Young’s modulus, mechanical strength, and strain at break of the materials to 1.79 GPa, 52.82 MPa, and 4.25% for blending 10 wt.% TPSS, respectively, and there are greater decreases of 1.44 GPa, 31.58 MPa, and 2.87% associated with increasing TPSS contents up to 40 wt.%, respectively. This result can be attributed to the greater brittleness of PLA/TPSS binary blends compared to the neat PLA, mainly caused by the thermodynamically immiscible aspect due to the inferior interfacial interaction between the hydrophilic TPSS and the hydrophobic PLA, as mentioned by various articles in most cases [44,45]. Afterwards, the PLA/20TPSS blend was specifically chosen for adding the modified rubber as a toughening agent by considering the stretchability and impact resistance of the binary blends. Figure 5b displays the stress–strain curves of PLA/20TPSS/MNR ternary blends with various MNR contents (1 to 10 wt.%). The addition of MNR in the binary blend provides the materials with the aspect of yielding and necking before the specimen fracture, indicating the transformation of the brittle-to-ductile fashion of the material. Moreover, there is a gradual increment in elongation at break by increasing the MNR in the PLA/TPSS binary blend system from 4.12% (PLA/20TPSS blends without rubber content) upwards to 4.34% (1 wt.% MNR), 10.27% (7 wt.% MNR), and 8.82% (10 wt.% MNR); however, the results see a concurrent reduction in tensile modulus and mechanical strength from 1.71 GPa and 52.49 MPa for PLA/20TPSS blends without rubber, respectively, down to 1.34 GPa and 32.05 MPa for the PLA/20TPSS/10MNR ternary blend. This finding reveals a significant improvement in the ductility of the brittle PLA binary blend by adding the modified rubber, which improves by approximately 112% and 149% the maximum extensibility for the PLA/20TPSS/7MNR ternary blend compared to the neat PLA and PLA/20TPSS blend without rubber, respectively. Meanwhile, the significant reduction in rigidity and strength of the PLA ternary blends can be attributed to the elastomeric nature of MNR added in the blend system, a similar behavior to the previous literature [33,46]. Additionally, a gradual drop in the modulus of the PLA ternary blend is consistent with the gradual reduction in hardness (Shore D) from approximately 78 to 69 Shore D by adding 1 wt.% MNR to 10 wt.% MNR contents.

**Figure 6:**
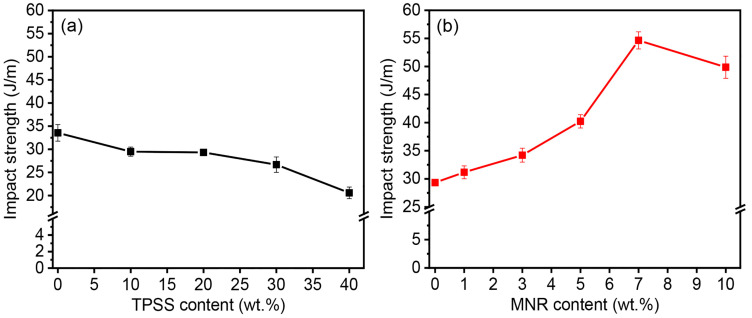
Impact strength of (**a**) PLA/TPSS binary blends with various TPSS contents and (**b**) PLA/20TPSS/MNR ternary blends with various MNR contents.

The impact strength, the capability to withstand a suddenly applied load, of the PLA binary and ternary blends were also investigated, as illustrated in Figure 6, and the relevant values are listed in Table 2. The addition of TPSS decreases the fracture resistance of the PLA material. The impact strength value reduces gradually from approximately 34 J/m for the neat PLA to 30 J/m (PLA/10TPSS blend) and then decreases downward to 21 J/m (PLA/40TPSS blend), as shown in Figure 6a. This phenomenon can be explained by the poor interfacial interaction between PLA and thermoplastic starch, which are relatively brittle, resulting in a substantial decline in the impact resistance of the binary blends [24]. Consequently, this work suggests the MNR as a renewable toughness modifier for the binary blend system. The PLA/20TPSS blend formulation was selected for toughening by the modified-rubber-containing hydroxyl group. Figure 6b demonstrates the impact strength of the PLA ternary blend by varying the rubber contents (1 to 10 wt.%). As anticipated, there is a substantial increment in the fracture resistance of the material from approximately 29 J/m (PLA/20TPSS binary blend) up to 55 J/m for the ternary blend containing 7 wt.% of rubber that slightly decreases to 50 J/m by adding 10 wt.% MNR. The maximum increment of the PLA/20TPSS/7MNR blend is about 62% and 90% improvement compared to the neat PLA and PLA/20TPSS binary blend. This finding can elucidate that the fracture energy obtained from an external load can be absorbed and dissipated by the dispersed elastomeric phase in the ternary blend; thus, the sudden breakage is delayed [47]. Moreover, the interfacial tension and surface energy between PLA and TPSS may be mitigated by adding the MNR, resulting in higher impact strength of the ternary blends than that of the PLA binary blends. It is probable that the hydroxyl group of the MNR can play a vital role in forming chemical and physical bonding with PLA and TPSS during the melt-mixing process, resulting in the material’s improved extensibility and fracture resistance.

#### 3.2.2. Morphological Aspects

**Figure 7:**
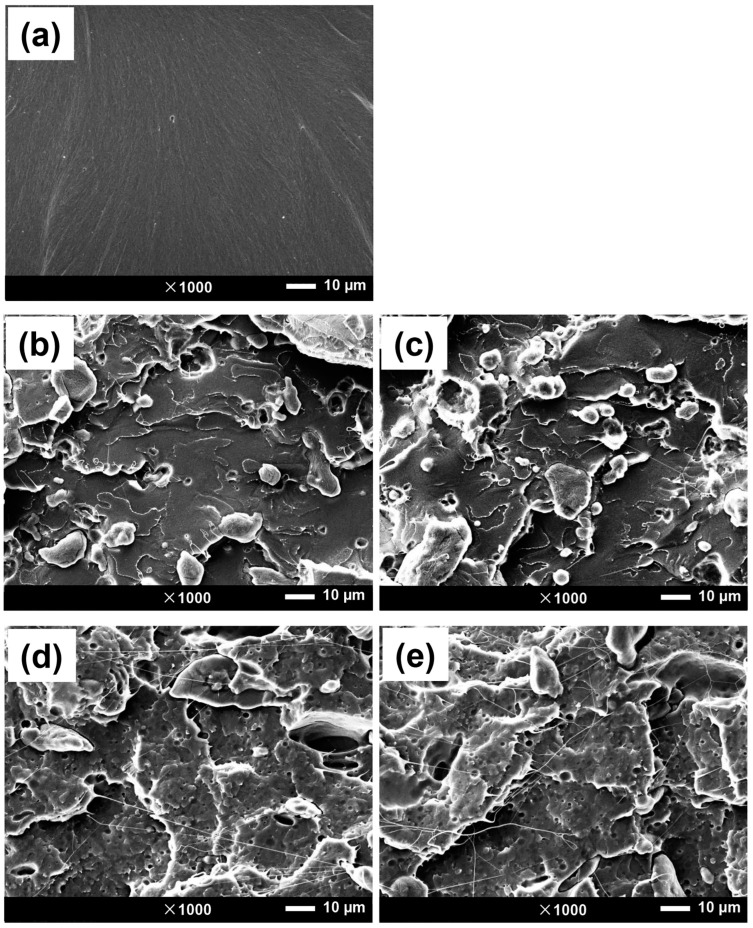
SEM images of the impact-fractured surface of (**a**) PLA, (**b**) PLA/20TPSS, (**c**) PLA/40TPSS, (**d**) PLA/20TPSS/3MNR, and (**e**) PLA/20TPSS/7MNR.

The notable improvement in stretchability and impact strength of the polymer blend and composite system is theoretically consistent with its microstructure. Figure 7 illustrates the impact-fractured surface of the PLA samples. The neat PLA shows a homogeneous and smooth fracture, revealing the typical rigid and brittle polymeric material. As anticipated, the addition of TPSS provides the PLA binary blends’ microstructure with the heterogeneous fracture and phase separation between the PLA matrix and the shapeless TPSS granules as a secondary phase. An obvious edge and aperture between the PLA matrix and TPSS granules (Figure 7b,c) assert the immiscible aspect, associated with the poor interfacial interaction of the two components. Subsequently, the addition of the MNR as a toughening agent, a third phase, in the PLA/20TPSS binary blend reveals the microvoids to be better dispersed and distributed throughout the fractured surface of the PLA ternary blends. White elastomeric microfibrils are also observed on the surface and seem to be the partial linkage between the PLA matrix and TPSS granules (Figure 7d,e). The elongated microfibrils reveal the MNR being stretched when the external force was applied, indicating sufficient interfacial adhesion between the PLA matrix and the dispersed MNR particles. Furthermore, the MNR plays a critical role in preventing the TPSS granule aggregation, resulting in a good distribution of TPSS in the PLA matrix. As a result, it can elucidate why the ductility of the materials is improved. 

**Figure 8:**
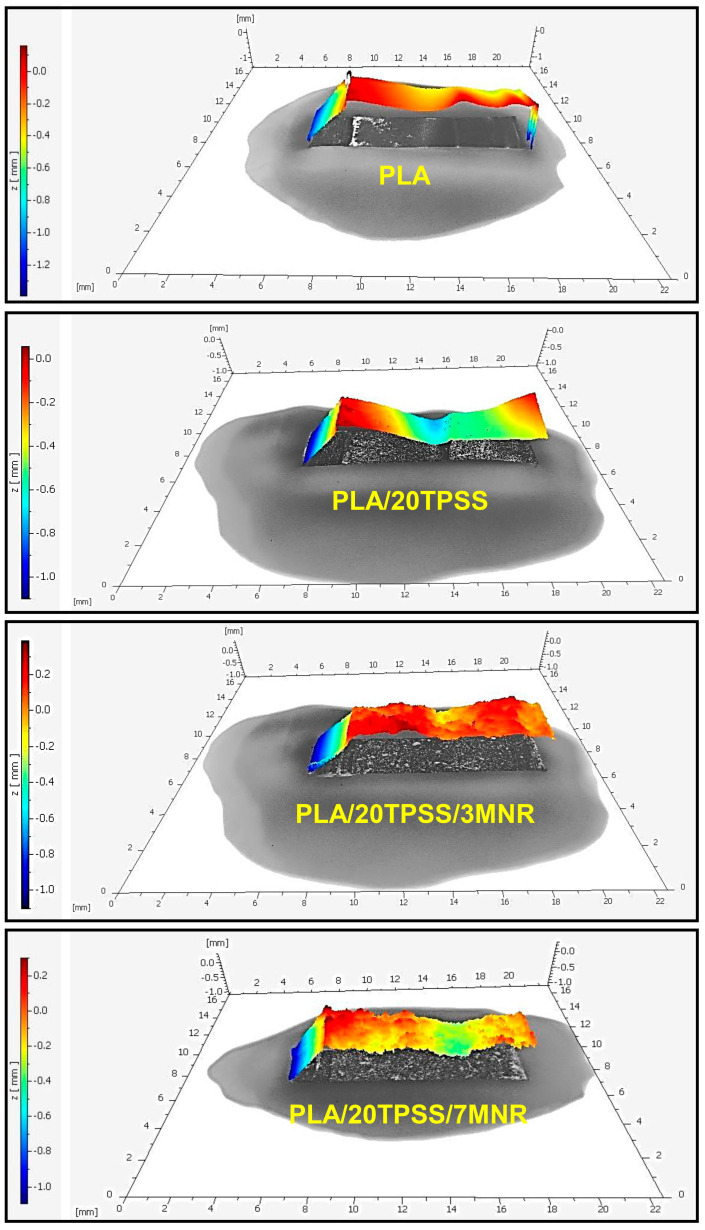
Three dimensional (3D) topographic color maps of the impact-fractured surfaces of PLA and its blends in the top view and the side view.

In addition, the improved toughness of the desired material can also be observed from the topography of the crack fracture surface. Generally, increasing the surface irregularity or roughness of the crack fracture indicates the improving interaction between the two or more components of polymer blends and composites, enhancing the mechanical properties of materials, especially impact strength [48,49]. Figure 8, therefore, visualizes the three-dimensional (3D) topographic color maps of the PLA and its blends, observing the surface fracture of the impact-fractured specimens. The fracture process is initiated from a crack tip and continuously propagated throughout the sample after an external load is suddenly applied. The topographic color map of the neat PLA reveals a smooth surface with a similar shade of the high-color scale across the surface fracture, similar to that observed in the SEM images. The addition of TPSS results in a relatively rough surface due to the protruding TPSS granules. The stress penetration during the crack process may probably run through the weak interface between the PLA matrix and the TPSS granules, leading to a deep crack surface consistent with the crack mechanism prediction. Afterwards, the presence of MNR as a toughening agent for the PLA/20TPSS binary blend exhibits broken fracture paths with the highly apparent rough surface, as obviously seen in the topographic map. This result indicates that the dispersed MNR phase may form a sufficient interfacial interaction with the PLA matrix and prevent TPSS granule aggregation, delaying the desired material’s crack fracture process. Additionally, the dispersed MNR phase, a green elastomeric material, can absorb and dissipate energy from an external source, improving the fracture resistance of the materials [33,50].

#### 3.2.3. Dynamic Mechanical Properties

**Figure 9:**
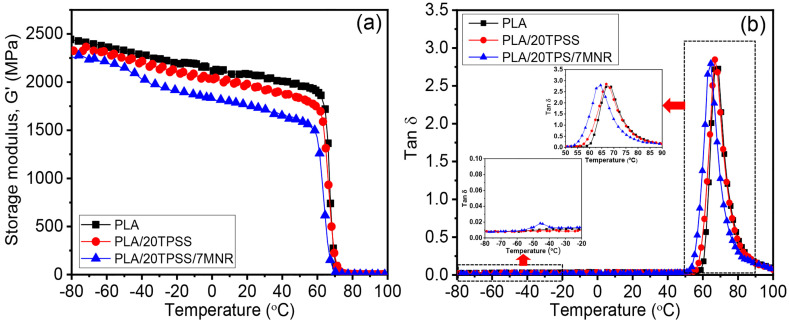
DMA thermograms of PLA, PLA/20TPSS binary blend, and PLA/20TPSS/7MNR ternary blend: (**a**) storage modulus and (**b**) tan δ.

The viscoelastic behavior of the samples was investigated, as shown in Figure 9. In this research, three PLA samples, including PLA, the PLA/20TPSS blend, and PLA/20TPSS/7MNR blend, were selected for a comparative study of PLA binary and ternary blends in terms of the storage modulus (G’) and loss tangent (tan δ) as a function of temperature. Generally, the G’ reveals the energy stored and recovered during the materials’ dynamic deformation or elastic response. The tan δ demonstrates the viscous behavior involving polymeric materials’ glass transition temperature (T_g_). The miscibility of polymer blends can be assessed from the shift in the T_g_ of the polymer matrix approach to the T_g_ of another phase [51]. As a result, the neat PLA shows the typical rigid material with the highest G’ value and T_g_ (~69 °C). The presence of TPSS decreases the stiffness of the binary blend without a change in the glass-transition relaxation behavior, as illustrated in the tan δ curve (Figure 9b). Subsequently, the addition of the MNR as a toughness modifier witnesses an apparent decline in the G’ value caused by the elastomeric nature of the modified rubber. This result is consistent with the previous reports on utilizing elastomer material in the PLA blends [27,48]. Furthermore, there is a significant shift in the T_g_ value of the ternary blend from approximately 69 °C for the neat PLA and the binary blend toward the lower temperature zone by about 5 °C. This finding may be attributed to the partial compatibility between the MNR and PLA matrix and probably the TPSS phase through the physical and chemical interaction during the melt-mixing process, resulting in the toughness improvement of the ternary blend.

#### 3.2.4. Thermal Properties

**Figure 10:**
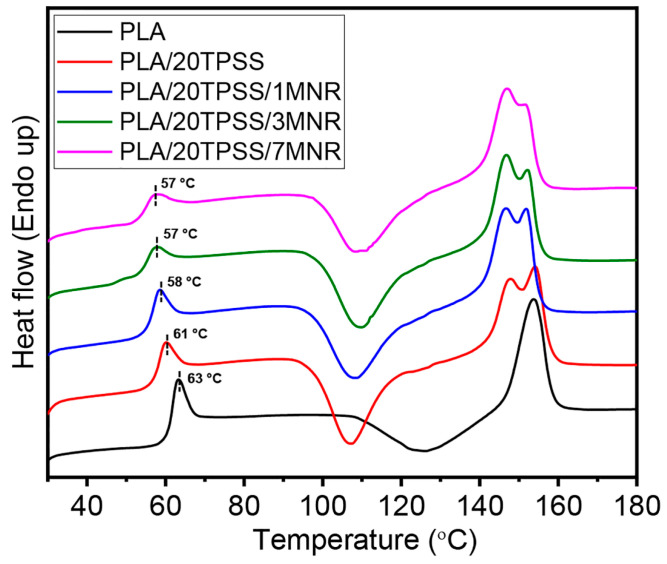
DSC thermograms of PLA, PLA/20TPSS binary blend, and PLA/20TPSS/MNR ternary blends at 1, 3, and 7 wt.% MNR.

The data of the thermal transition behavior of PLA and its blends were evaluated as summarized in Table 3, and the examples of DSC thermograms are shown in Figure 10. The neat PLA exhibits a typical semi-crystalline polymer with the endothermic peak of the glass-transition temperature (T_g_) at approximately 63 °C, the broad exothermic peak of the cold crystallization temperature (T_cc_) at about 126 °C, and the single endothermic peak of the melting temperature (T_m_) at around 154 °C, having a low crystallinity level of about 6.72%. The presence of TPSS ranging between 10 wt.% and 40 w.% provides the binary blends with a slight reduction in the T_g_ values to about 61 °C and a significant decline in the T_cc_ values downward approximately 109 °C. This result reveals that the TPSS may probably act as a plasticizer to increase the segmental chain motion of PLA in the blends, resulting in a decrement in the T_g_ and T_cc_ values of PLA binary blends [27]. Generally, the shift in T_g_ and T_cc_ values closely relates to the polymeric material’s segmental chain motion during heating [52]. Moreover, the PLA binary blend system shows two distinct endothermic peaks at around 146 °C to 148 °C and 152 °C to 154 °C, attributing to the change in lamellar rearrangement and recrystallization of the PLA crystalline structure [53]. Subsequently, the addition of the MNR as a toughness modifier in the binary blend shows an evident decline in the T_g_ values by approximately 3–4 °C and about 5–6 °C compared to the PLA/20TPSS blend and the neat PLA, respectively; meanwhile, it reveals a slight increase in the T_cc_ value, as seen in Figure 10. These phenomena can elucidate the formation of partial interaction of the dispersed MNR and PLA matrix (and the TPSS phase), consistent with the DMA result. As for the melting behavior, all PLA ternary blends show similar aspects to the binary blends system. Considering the crystallinity level, the crystallinity of PLA (6.72%) was reduced by adding the TPSS downward 2.77% by adding 40 wt.% TPSS. The addition of 1 wt.% MNR into the PLA/20TPSS binary blend reveals a slight increment in the crystallinity degree of the material (4.56%), likely indicating that the modified rubber may act as a nucleating agent in this ternary blend formulation. However, adding higher rubber contents tends to reduce the ternary blends’ crystallinity level, as summarized in Table 3.

#### 3.2.5. Water Absorption

**Figure 11:**
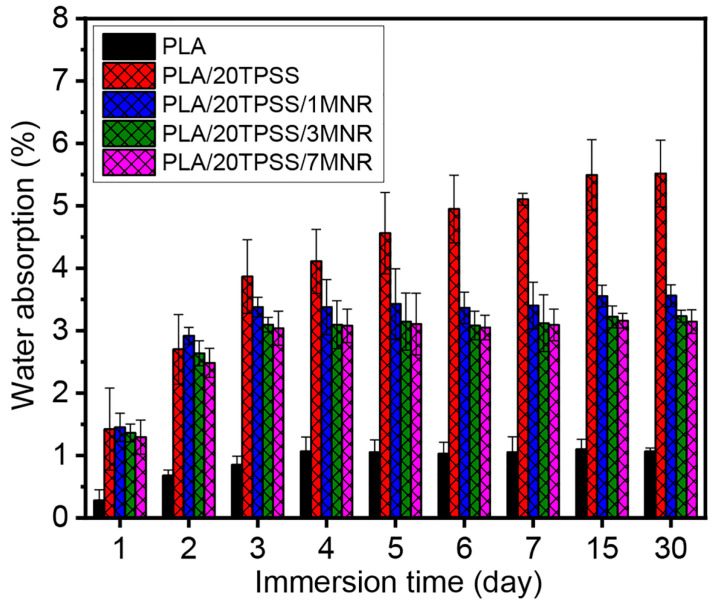
Water absorption of PLA, PLA/20TPSS binary blend, and PLA/20TPSS/MNR ternary blend with various MNR contents (1, 3, and 7 wt.%) at various immersion times.

**Figure 12:**
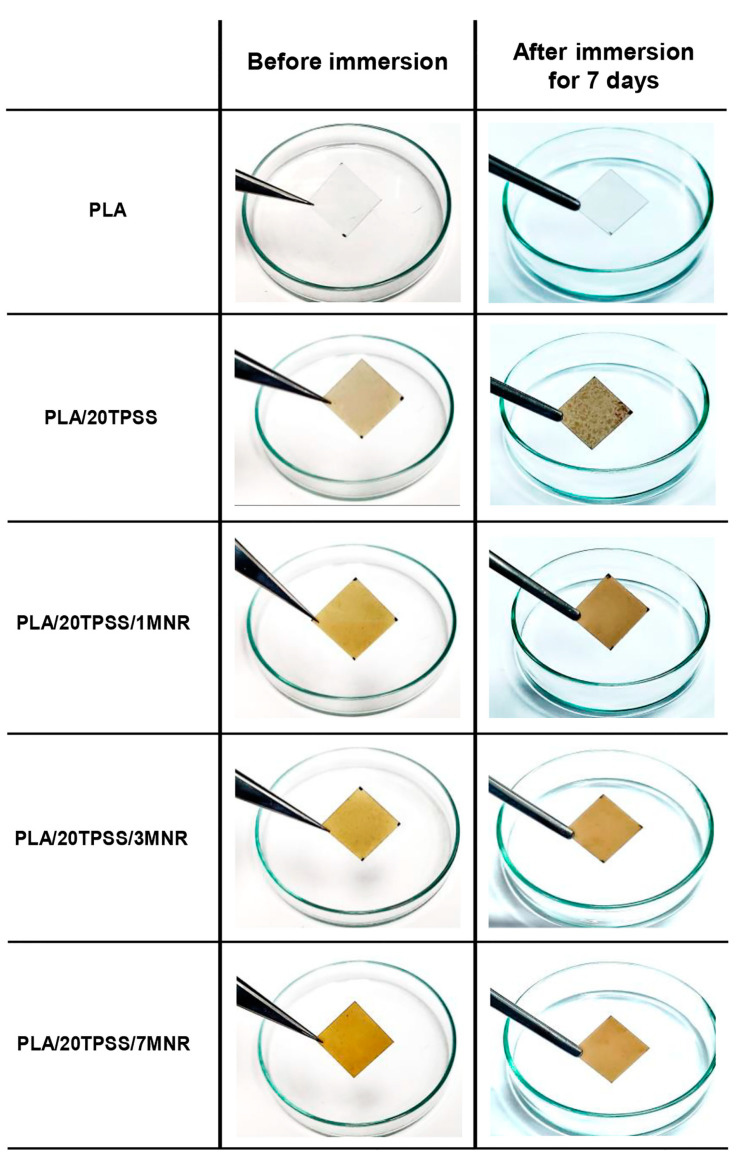
Physical appearance of PLA sheets before and after immersion in the water for 7 days.

The water resistance of the neat PLA and its blends was assessed in terms of water absorption with time intervals (1 to 30 days), as illustrated in Figure 11. As a result, the water absorption capacity increases with an escalation of immersion times, ultimately levelling off after a certain period. The neat PLA possesses the lowest water absorption capacity, less than approximately 1%, with a stable value after four days throughout the period, indicating that PLA is a low-hydrophilic polymeric material [54,55]. Afterwards, the moisture absorption capacity of the material increases rapidly upward by 3 to 4% by adding the TPSS within the first few days of immersion, involving the effect of the hydrophilic nature of TPSS via the existence of the hydroxyl functional groups available for interaction with the water molecules. Considering the PLA ternary blends, this result indicates that the stable point of moisture absorption value tends to decrease by adding the modified rubber. This can elucidate the impact of the hydrophobic feature of natural rubber-based material added in the PLA/TPSS binary system, resulting in the improved water resistance of the material. Additionally, the physical appearance of sample sheets before and after immersion in the water for 7 days was observed, as presented in Figure 12. As anticipated, the neat PLA still shows a typical transparent sheet, while the binary blend demonstrates the brown spots distributed throughout the sheet, involving the mold growth in the sample after the immersion test. The ternary blends do not show any changes in the sheet’s physical appearance compared to the sample before the immersion test. This phenomenon is consistent with the water absorption capacity of the PLA blend samples.

#### 3.2.6. Biodegradability

**Figure 13:**
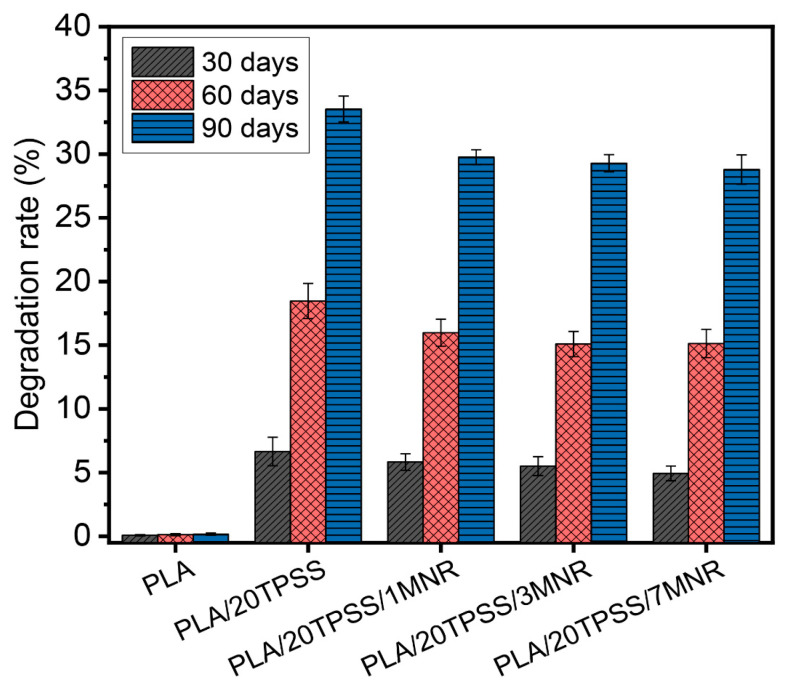
Degradation rate of PLA and its blends measured by weight loss after the soil burial test at various burial times (30, 60, and 90 days).

Figure 13 displays the biodegradability of the PLA samples measured by the weight loss after the soil burial test at various times (30, 60 and 90 days). As expected, the neat PLA weight remained constant without any physical change throughout the three months of the soil burial test period, and the degradation rate was elevated with an increment of the soil burial time. The incorporation of 20 wt.% TPSS reveals a rapid increment in the weight loss rate of the PLA binary blends, which are observed at approximately 7%, 18%, and 33% at burial times of 30, 60, and 90 days, respectively. This result is similar to the previous reports in which the PLA/starch blends generally had a higher weight loss rate than the neat PLA [27]. Furthermore, the presence of TPSS as a hydrophilic material can contribute to the moisture diffusion into the PLA matrix, likely resulting in the acceleration of the PLA chains’ hydrolysis [56]. In the cases of the ternary blend system, the addition of MNR in the PLA/20TPSS blend exhibits a slight decline in the degradation rate. This result involves the effect of the hydrophobic nature of the modified rubber added in the binary blend system, causing a slight delay in the degradability of the prepared material; however, the ternary blends in this work showcase the accelerating degradability and improvement in toughness of the prepared material when compared to that of the neat PLA.

**Figure 14:**
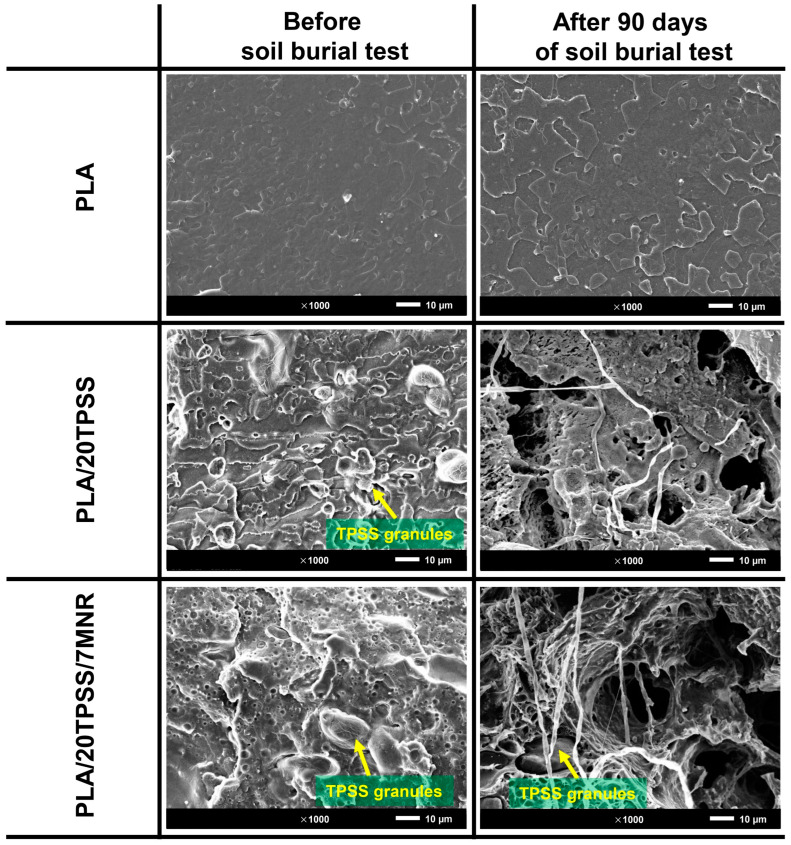
SEM images of the PLA sheet samples before and after the soil burial test for 90 days.

Moreover, the microscopic observation of the sample sheets before and after the soil burial test was visualized, as presented in Figure 14. As a result, the morphological fashion of the neat PLA after the soil burial for 90 days does not change, relatively, when compared with the neat PLA before the burial test, clearly showing the flat surface without any deterioration. The degradation of the PLA/20TPSS binary blend at the microstructure level is clearly detected, with an occurrence of many holes observed without any TPSS granules, indicating that biodegradation started from the hydrophilic TPSS phase, contributing to the moisture diffusion in the sample sheet and promoting the hydrolysis process of the PLA matrix. Moreover, the hydrophilic aspect of the TPSS makes the PLA sensitive to humidity and can act as an excellent nutrient source for the colonization of microorganisms [27,57], observing the fungi fibers grown within the sample, resulting in the deterioration of the PLA materials. Subsequently, the addition of the modified rubber to toughen the binary blend system displays a similar aspect, exhibiting a large number of holes and fungi fibers; nevertheless, the TPSS granules were still observed in the deteriorating sample, consistent with the slighter degradation rate of the ternary blend as aforementioned. Consequently, the development of PLA material, with its improving toughness and accelerating biodegradability using pineapple stem starch, a massive source of agricultural waste, and natural rubber, an eco-friendly elastomer and renewable material, was successfully prepared in this research work to fulfil the limitation gap of PLA’s application.

## 4. Conclusions

The development of desired PLA materials with enhanced impact strength and accelerated biodegradability was achieved through the successful utilization of thermoplastic pineapple stem starch (TPSS) and modified natural rubber (MNR) in a melt-blending process. The addition of TPSS to prepare the PLA/TPSS binary blend resulted in a reduction in tensile, impact, and water-resistant properties; however, it exhibited much-improved biodegradability. With the inclusion of MNR in the binary blend, the resulting PLA ternary blends demonstrated improved stretchability and fracture resistance, showcasing suitable modulus and mechanical strength for various applications. Notably, the impact strength increased by approximately 62% and 90% compared to neat PLA and the PLA/20TPSS binary blend with the addition of only 7 wt.% MNR. This enhancement can be attributed to improved interfacial interaction between the PLA matrix and the TPSS phase, facilitated by MNR as a toughness modifier, as confirmed by dynamic mechanical analysis, thermal transition behavior, phase morphology, and topography. Furthermore, the prepared ternary blends exhibited superior water resistance compared to binary blends, coupled with proper biodegradability, rendering them suitable for diverse applications. Leveraging pineapple stem starch, derived from substantial agricultural waste, and natural rubber, an eco-friendly elastomer and renewable resource, presents an innovative and sustainable approach to address the challenges associated with PLA for use in plastic packaging applications. This research contributes to the potential and effective avenues for advancing economic models while mitigating carbon footprint consequences, representing a step towards a more sustainable future.

## Figures and Tables

**Table 1:** Formulation of PLA/TPSS and PLA/TPSS toughened by MNR.

Sample Code	PLA (wt.%)	TPSS (wt.%)	MNR (wt.%)
PLA	100	0	0
PLA/10TPSS	100	10	-
PLA/20TPSS	100	20	-
PLA/30TPSS	100	30	-
PLA/40TPSS	100	40	-
PLA/20TPSS/1MNR	100	20	1
PLA/20TPSS/3MNR	100	20	3
PLA/20TPSS/5MNR	100	20	5
PLA/20TPSS/7MNR	100	20	7
PLA/20TPSS/10MNR	100	20	10

**Table 2:** Tensile properties, impact strength, and hardness (Shore D) of PLA/TPSS binary blends and PLA/TPSS/MNR ternary blends.

Sample Code	Tensile Properties			Impact Strength (J/m)	Hardness (Shore D)
	*E* * (GPa)	σ ** (MPa)	ε ***(%)		
PLA	2.1 ± 0.1	73.18 ± 4.37	4.84 ± 0.35	33.55 ± 1.80	80 ± 1
PLA/10TPSS	1.8 ± 0.2	52.82 ± 3.86	4.25 ± 0.44	29.49 ± 0.99	81 ± 1
PLA/20TPSS	1.7 ± 0.2	52.49 ± 3.66	4.12 ± 0.29	29.32 ± 0.46	82 ± 1
PLA/30TPSS	1.7 ± 0.1	40.63 ± 4.79	3.28 ± 0.32	26.67 ± 1.67	84 ± 2
PLA/40TPSS	1.4 ± 0.2	31.58 ± 3.14	2.87 ± 0.31	20.58 ± 1.26	84 ± 2
PLA/20TPSS/1MNR	1.7 ± 0.2	51.29 ± 3.66	4.34 ± 0.26	31.16 ± 1.15	78 ± 1
PLA/20TPSS/3MNR	1.7 ± 0.2	37.51 ± 2.37	6.44 ± 0.43	34.21 ± 1.25	75 ± 2
PLA/20TPSS/5MNR	1.6 ± 0.1	33.88 ± 1.29	8.01 ± 0.49	40.23 ± 1.18	74 ± 2
PLA/20TPSS/7MNR	1.6 ± 0.2	33.11 ± 1.07	10.27 ± 0.47	54.65 ± 1.53	72 ± 2
PLA/20TPSS/10MNR	1.3 ± 0.2	32.05 ± 1.43	8.82 ± 0.46	49.86 ± 1.97	69 ± 2

* Tensile modulus, ** tensile strength, *** elongation at break.

**Table 3:** DSC data of PLA, PLA/TPSS binary blends, and PLA/TPSS/MNR ternary blends.

Sample Code	T_g_ (°C)	T_cc_ (°C)	ΔH_cc_ (J/g)	T_m_ (°C)	ΔH_m_ (J/g)	*X*_c_ (%)
PLA	62.7	126.4	11.5	153.8	17.76	7
PLA/10TPSS	61.1	108.8	19.5	148.1, 154.0	22.95	4
PLA/20TPSS	61.0	108.9	19.3	146.8, 153.1	22.47	4
PLA/30TPSS	61.0	108.2	19.2	146.4, 152.8	22.09	4
PLA/40TPSS	61.1	109.9	17.3	146.3, 152.0	19.15	3
PLA/20TPSS/1MNR	58.2	109.2	19.1	146.5, 152.6	22.83	5
PLA/20TPSS/3MNR	58.2	110.4	20.2	146.5, 151.9	23.54	4
PLA/20TPSS/5MNR	57.4	110.7	21.2	146.1, 152.1	23.04	4
PLA/20TPSS/7MNR	57.4	110.7	19.7	146.7, 151.7	22.59	4
PLA/20TPSS/10MNR	57.3	110.2	19.6	146.6, 152.1	22.17	4

## Data Availability

The data presented in this study are available on request from the corresponding author.
